# Sodium butyrate enhances STAT 1 expression in PLC/PRF/5 hepatoma cells and augments their responsiveness to interferon-α

**DOI:** 10.1038/sj.bjc.6690413

**Published:** 1999-05

**Authors:** W-C Hung, L-Y Chuang

**Affiliations:** School of Technology for Medical Sciences and Department of Biochemistry, Kaohsiung Medical College, No. 100, Kaohsiung, 807, Taiwan, Republic of China

**Keywords:** sodium butyrate, interferon, hepatoma, cyclin D1, p21^WAF-1^, retinoblastoma protein, apoptosis

## Abstract

Although interferon-α (IFN-α) has shown great promise in the treatment of chronic viral hepatitis, the anti-tumour effect of this agent in the therapy of liver cancer is unclear. Recent studies have demonstrated that differentiation-inducing agents could modulate the responsiveness of cancer cells to IFN-α by regulating the expression of signal transducers and activators of transcription (STAT) proteins, a group of transcription factors which play important roles in the IFN signalling pathway. We have reported that sodium butyrate is a potent differentiation inducer for human hepatoma cells. In this study, we investigated whether this drug could regulate the expression of STAT proteins and enhance the anti-tumour effect of IFN-α in hepatoma cells. We found that sodium butyrate specifically activated STAT1 gene expression and enhanced IFN-α-induced phosphorylation and activation of STAT1 proteins. Co-treatment with these two drugs led to G1 growth arrest, accompanied by down-regulation of cyclin D1 and up-regulation of p21^WAF-1^, and accumulation of hypophosphorylated retinoblastoma protein in hepatoma cells. Additionally, internucleosomal DNA fragmentation, a biological hallmark of apoptosis, was detected in hepatoma cells after continuous incubation with a combination of these two drugs for 72 h. Our results show that sodium butyrate potently enhances the anti-tumour effect of IFN-α in vitro and suggest that a rational combination of these two drugs may be useful for the treatment of liver cancer. © 1999 Cancer Research Campaign


British Journal of Cancer (1999) **80**(5/6), 705–710
© 1999 Cancer Research Campaign
Article no. bjoc.1998.0413

### Sodium butyrate enhances STAT 1 expression in PLC/PRF/5 hepatoma cells and augments their responsiveness to interferon-α

**W-C Hung[1] and L-Y Chuang[2]**

[1]School of Technology for Medical Sciences and [2]Department of Biochemistry, Kaohsiung Medical College, No. 100, Shih-Chuan 1st Road, Kaohsiung 807, Taiwan, Republic of China

**Summary** Although interferon-α (IFN-α) has shown great promise in the treatment of chronic viral hepatitis, the anti-tumour effect of this agent in the therapy of liver cancer is unclear. Recent studies have demonstrated that differentiation-inducing agents could modulate the responsiveness of cancer cells to IFN-α by regulating the expression of signal transducers and activators of transcription (STAT) proteins, a group of transcription factors which play important roles in the IFN signalling pathway. We have reported that sodium butyrate is a potent differentiation inducer for human hepatoma cells. In this study, we investigated whether this drug could regulate the expression of STAT proteins and enhance the anti-tumour effect of IFN-α in hepatoma cells. We found that sodium butyrate specifically activated STAT1 gene expression and enhanced IFN-α-induced phosphorylation and activation of STAT1 proteins. Co-treatment with these two drugs led to G1 growth arrest, accompanied by down-regulation of cyclin D1 and up-regulation of $p21^{WAF-1}$, and accumulation of hypophosphorylated retinoblastoma protein in hepatoma cells. Additionally, internucleosomal DNA fragmentation, a biological hallmark of apoptosis, was detected in hepatoma cells after continuous incubation with a combination of these two drugs for 72 h. Our results show that sodium butyrate potently enhances the anti-tumour effect of IFN-α in vitro and suggest that a rational combination of these two drugs may be useful for the treatment of liver cancer.

**Keywords:** sodium butyrate; interferon; hepatoma; cyclin D1; $p21^{WAF-1}$; retinoblastoma protein; apoptosis

Interferons (IFNs) are cytokines that are involved in the regulation of diverse cellular functions, including modulation of immune responses, inhibition of proliferation, and induction of resistance to viral and bacterial infection (Gutterman, 1994). IFNs employ a unique signalling pathway for rapid induction of gene expression by utilizing two groups of critical elements to transduce the signals from cell surface receptor to nuclear events. The first is the janus family kinases (JAKs), a group of tyrosine kinases containing JAK1, JAK2, JAK3 and TYK2, and the second is STAT (signal transducers and activators of transcription) proteins, a group of latent cytoplasmic transcription factors which are activated by phosphorylation (Darnell, 1997; Pellegrini and Dusanter-Fourt, 1997). Upon IFN stimulation, receptor-associated JAKs are phosphorylated and activated and these kinases, in turn, phosphorylate and activate STAT proteins which then translocate from cytoplasm to nucleus and direct transcriptional activation of various effector genes. The essential role of JAKs and STATs in IFN signalling pathway has been demonstrated by the IFN-resistant phenotypes of cells lacking the JAK or STAT gene products (Muller et al, 1993; Xu et al, 1994).

Recent studies have shown that IFNs are the most promising anti-viral agents for the treatment of patients with chronic hepatitis B. These cytokines have been reported to suppress hepatitis B virus (HBV) enhancer activity, reduce virus replication and activate hepatocellular gene expression in cultured liver cells (Tur-Kaspa et al, 1990; Zhang and McLachlan, 1994). Additionally, results from clinical trials also demonstrated that IFNs caused inhibition of HBV replication and reduction of hepatitis B surface antigen (HBsAg) expression (Alexander et al, 1987; Korenman et al, 1991). These studies suggest that IFNs are useful anti-viral drugs for the treatment of patients with viral hepatitis. However, the use of IFNs in the therapy of liver cancer seems discouraging. Although several studies have shown that IFNs exerted growth-inhibitory effects on human hepatoma cells (Lai et al, 1989; Boue et al, 1990), other works showed that patients with advanced hepatocellular carcinoma were resistant to IFNs (Nair et al, 1985; Sachs et al, 1985). However, the molecular basis of this resistance is not clear.

Recent studies have indicated that the responses of cells to IFNs could be modulated by regulating the expression or the activity of JAKs and STATs. It has been found that elevated activity of JAKs enhanced the anti-tumour effects of IFNs to lymphoblastoid cells (Lee et al, 1997). Similarly, induction of STATs gene expression by retinoic acids in IFN-unresponsive breast cancer cells and myeloid leukaemia cells permitted growth inhibition by IFNs (Kolla et al, 1996; Weihua et al, 1997). These data suggest that the resistance of cancer cells to IFNs may be due to a relative deficiency of IFN signalling molecules and argue that overexpression of these signalling molecules may restore the responsiveness of cancer cells to IFNs. Among the drugs

*Received 17 July 1998*
*Revised 7 November 1998*
*Accepted 10 November 1998*

*Correspondence to:* W-C Hung examined, differentiation-inducing agents were found to be effective both in enhancing STATs expression and in promoting IFN-α-induced cell growth arrest in different tumour types (Goto et al, 1996; Pelicano et al, 1997). We have previously demonstrated that sodium butyrate, a natural fermentation product of colonic bacterial flora, induced differentiation in PLC/PRF/5 hepatoma cells (Hung et al, 1995). In this study, we investigated whether sodium butyrate modulated the expression of STATs in PLC/PRF/5 cells and altered the sensitivity of these cells to IFN-α.

### MATERIALS AND METHODS

### Reagents

Human recombinant IFN-α was kindly provided by Hoffmann-La Roche Inc. Antibodies against the members of the STAT gene family (STAT1–6) were obtained from Transduction Laboratories (Lexington, KY, USA) and monoclonal antibodies against human cyclin D1 and p21$^{WAF-1}$ were purchased from Calbiochem (San Diego, CA, USA). Anti-human retinoblastoma protein (pRB) antibody was obtained from Pharmingen (San Diego, CA, USA) and sodium butyrate and other chemicals were purchased from Sigma (St Louis, MO, USA).

### Cell culture and treatment

PLC/PRF/5 cells were cultured in Dulbecco's modified Eagle medium: nutrient mixture F12 (DMEM/F12) supplemented with 10% heat-inactivated fetal calf serum (FCS) in a 5% carbon dioxide incubator at 37°C. Cells were grown in 10% FCS medium to subconfluence and then incubated in medium containing sodium butyrate (1 mM) or IFN-α (200–2000 IU ml$^{-1}$) alone, or a combination of these two drugs for different time intervals, and were harvested for analysis.

### Analysis of cell cycle phase by flow cytometry

The drug-treated cells were trypsinized, washed with phosphate-buffered saline (PBS) and fixed in 95% ethanol at − 20°C for 1 h. Following collection, cells were resuspended in 400 µl of PBS containing 1 mg ml$^{-1}$ RNAase and 0.5% Triton X-100, incubated at room temperature for 1 h and stained with 50 µg ml$^{-1}$ of propidium iodide for another 30 min. Fluorescence emitted from the propidium iodide–DNA complex was analysed by FACScan flow cytometry (Becton Dickinson) and cell cycle distribution was calculated with the CellFit software.

### Immunoprecipitation and immunoblotting

After treatment, cells were washed with ice-cold PBS and harvested in a lysis buffer (50 mM Tris–HCl, pH 7.4, 150 mM, sodium chloride, 5 mM EDTA, 1% Triton X-100, 50 mM sodium fluoride (NaF), 1 mM sodium orthovanadate, 1 mM phenylmethylsulphonyl fluoride, 2 µg ml$^{-1}$ pepstatin A, 2 µg ml$^{-1}$ leupeptin and 1 µg ml$^{-1}$ aprotinin) for 20 min on ice and centrifuged at 12 000 ***g*** for 20 min. Protein concentration of lysates was determined by using a Bio-Rad protein assay kit. For immunoprecipitation, normalized volumes of extracts containing 500 µg protein were incubated for 4 h at 4°C with specific antibodies. Immunocomplexes were collected following incubation with protein G-agarose beads at 4°C for another 1 h. The beads were washed three times with lysis buffer and the immunocomplexes were eluted by boiling in the Laemmli sample buffer. Equal volumes of eluted immunocomplexes were then subjected to immunoblotting analysis. For immunoblotting, eluted immunocomplexes or cell lysates (50 µg protein per sample) were subjected to sodium dodecyl sulphate-polyacrylamide gel electrophoresis (SDS-PAGE). Proteins were transferred to nitrocellulose filters and the blots were blocked overnight at 4°C in 5% non-fat milk in TBST (20 mM Tris–HCl, pH 7.4, 137 mM sodium chloride and 0.05% Tween-20). The blots were washed in TBST and incubated with various antibodies for 2 h at room temperature. After extensively washing in TBST buffer, peroxidase-conjugated secondary antibody was added in blocking buffer for another 1 h and the blots were developed by using the ECL chemiluminescence system (Amersham). The signal intensities of immunoblots were quantified by densitometric scanning.

### Electrophoretic mobility shift assay

Nuclear extract preparation and electrophoretic mobility shift assay EMSA analysis was performed as previously described (Decker et al, 1991). In brief, a double-stranded oligonucleotide corresponding to the GAS element found within the promoter of the guanylate-binding protein (GBP) gene was synthesized and end-labelled using polynucleotide kinase and [γ-$^{32}$P]-adenosine 5′-triphosphate (ATP) and incubated with nuclear extracts for 20 min at room temperature. The oligonucleotide–protein complexes were fractionated on a 6% non-denatured polyacrylamide gel and detected by autoradiography. For competition, a 20-fold excess of unlabelled oligonucleotides or 1 µg anti-STAT 1 or anti-c-Jun antibodies were added to reaction mixture.

### Morphological analysis of apoptotic cells

Cells were cultured in 6-well plates in complete medium containing different combinations of drugs for 72 h. After incubation, detached cells were pelleted by centrifugation and attached cells were harvested by trypsinization. Cells were pooled and collected at 3000 ***g*** for 10 min and washed twice with PBS. Cells were fixed with 3% paraformaldehyde in PBS for 15 min at room temperature and stained with Hoechst 33258 dye for another 15 min at room temperature. Stained cells were placed on glass slides and observed under a fluorescence microscope. For each sample, 500 cells were counted and apoptotic cells with condensed chromatin fragments were scored and expressed as a percentage of total cell number measured.

### DNA extraction and agarose gel electrophoresis

After drug incubation, cells were harvested and genomic DNA was extracted and analysis of DNA fragmentation on agarose gel was performed as described previously (Hung and Chuang, 1996).

### RESULTS

### Specific induction of STAT1 expression by sodium butyrate in PLC/PRF/5 cells

Our previous results have indicated that sodium butyrate is a potent differentiation-inducing agent to PLC/PRF/5 hepatoma cells. This agent effectively inhibited α-fetoprotein secretion,

© Cancer Research Campaign 1999

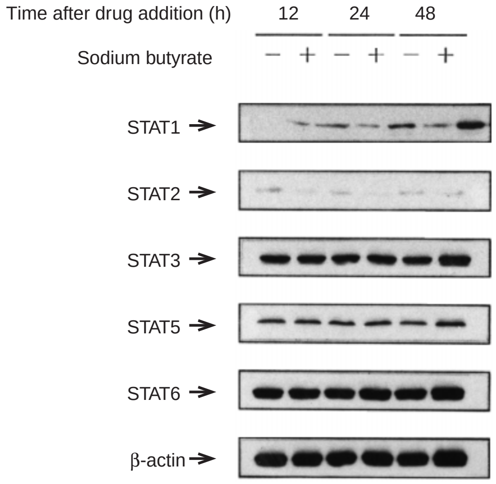

**Figure 1** Regulation of STATs expression by sodium butyrate in PLC/PRF/5 cells. Cells were treated with 1 mM sodium butyrate for different time intervals and harvested in a lysis buffer. In each lane, 50 μg protein of each preparation were separated by SDS-PAGE, transferred to nitrocellulose membrane and subjected to immunoblotting analysis. The blots were probed with specific anti-STAT antibodies and developed by using a chemiluminescence substrate kit

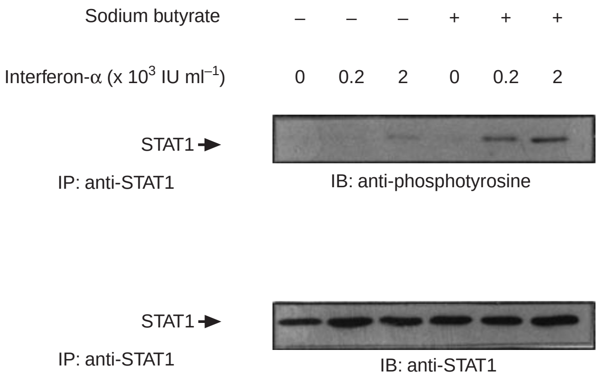

**Figure 2** IFN-α-induced phosphorylation of STAT1 was enhanced by sodium butyrate. Cells were cultured in the absence or presence of 1 mM sodium butyrate for 2 days and then stimulated with different concentrations of IFN-α for 30 min. Whole cell lysates were isolated and protein concentrations were determined. For each sample, 300 μl of whole cell lysates containing 500 μg of cellular proteins was immunoprecipitated with 1 μg anti-STAT1 monoclonal antibody at 4°C for 4 h. Immunocomplexes were collected by adding the protein G-agarose beads and eluted by boiling in the Laemmli sample buffer. Immunoprecipitates were separated by SDS-PAGE, transferred to nitrocellulose membranes and probed with anti-STAT1 or antiphosphotyrosine antibodies. IP: immunoprecipitation. IB: immunoblotting

**Table 1** Alteration of protein levels of STATs in PLC/PRF/5 cells after sodium butyrate treatment

| | Ratio (STAT intensity/β-actin intensity) | | |
|---|---|---|---|
| STATs | − sodium butyrate | + sodium butyrate | Fold increase |
| STAT1 | 0.20 ± 0.05 | 0.45 ± 0.05[a] | 2.25 |
| STAT2 | 0.13 ± 0.04 | 0.11 ± 0.03 | 0.85 |
| STAT3 | 0.45 ± 0.09 | 0.50 ± 0.17 | 1.11 |
| STAT5 | 0.29 ± 0.08 | 0.33 ± 0.14 | 1.13 |
| STAT6 | 0.84 ± 0.15 | 0.90 ± 0.12 | 1.07 |

Cells were cultured in the absence or presence of 1 mM sodium butyrate for 48 h and immunoblotting analysis was performed as described in Figure 1. Signal intensities of STATs were quantified by densitometric scanning and then normalized with signal intensities of β-actin. The values represent mean ± s.d. of three different experiments. Significance of differences between cells treated without or with 1 mM sodium butyrate was calculated by using Student's *t*-test, [a]$P < 0.01$.

increased albumin expression and caused PLC/PRF/5 cells to acquire in vitro properties which were more consistent with well-differentiated cells in a dose-dependent manner (Hung et al, 1995). Since sodium butyrate at 1 mM induced phenotypic changes in PLC/PRF/5 cells as described above and did not show any cytotoxicity in these cells after a 3-day treatment, we therefore routinely used 1 mM sodium butyrate to treat cells in this work. Similar results were observed when higher concentrations of sodium butyrate were added (data not shown). We first asked whether sodium butyrate modulated the expression of STATs in this cell line. As shown in Figure 1, PLC/PRF/5 cells cultured in 10% FCS medium constantly expressed all STATs, except STAT4, proteins. However, significant differences between the expression levels of STATs in this cell line were found. PLC/PRF/5 cells expressed a large amount of STAT6 and a relatively smaller amount of STAT1, STAT3 and STAT5, while very little amount of STAT2 was detected. Lack of STAT4 in PLC/PRF/5 cells is not surprising because the expression of STAT4 has been reported to be limited to thymus, spleen and testis (Yamamoto et al, 1994). The difference between the expression of STATs was further confirmed by probing the membrane with anti-β-actin antibody to verify equal loading of proteins in each lane. Figure 1 also shows that sodium butyrate did not regulate the expression of STAT 3, 5, 6 in PLC/PRF/5 cells after a 48-h incubation and this agent slightly decreased the protein level of STAT2 under the same experimental conditions. Conversely, the expression of STAT1 was significantly (a 2.25-fold increase) enhanced after treatment with sodium butyrate for 2 days. Also, the difference in protein levels of STATs detected by immunoblotting is not due to the difference of quality of the used antibodies, because positive controls (lystates of Jurkat human leukaemia cells or rat testis provided by the manufacturer of antibodies) were included in our immunoblotting assays to confirm the results. Furthermore, the relative signal intensities of STATs (normalized with the signal intensities of β-actin) after sodium butyrate treatment are shown in Table 1.

### Sodium butyrate enhances the activation of STAT1 by IFN-α

Since sodium butyrate significantly up-regulates the expression of STAT1, we investigated whether IFN-α-induced phosphorylation and activation of STAT1 was enhanced in sodium butyrate-pretreated PLC/PRF/5 cells. The phosphorylation status of STAT1

**Table 2** Effect of sodium butyrate and IFN-α on cell cycle progression in PLC/PRF/5 cells analysed by flow cytometric analysis

| | Cell cycle distribution (%) | | |
|---|---|---|---|
| **Treatment** | **G1** | **S** | **G2/M** |
| Control (no addition) | 43.2 | 41.7 | 15.1 |
| Sodium butyrate (1 mM) | 53.9 | 31.6 | 14.5 |
| IFN-α (2000 IU ml$^{-1}$) | 61.3 | 25.7 | 13.0 |
| Sodium butyrate + IFN-α | 83.8 | 7.1 | 9.1 |

© Cancer Research Campaign 1999 

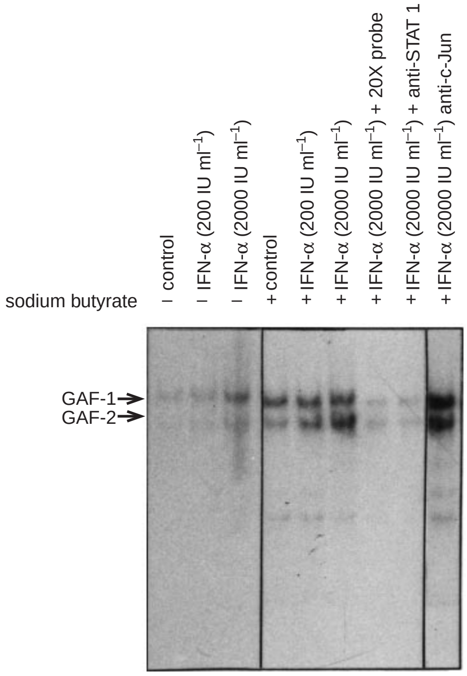

**Figure 3** Sodium butyrate enhancement of STAT1 activation by IFN-α in PLC/PRF/5 cells. Cells were first cultured in the absence or presence of 1 mM sodium butyrate for 2 days and stimulated with different concentrations of IFN-α for 30 min. Nuclear extracts were prepared and electrophoretic mobility shift assays were performed as described in Materials and Methods. For competition experiments, specific unlabelled probes (20 X), anti-STAT1 or anti-c-Jun antibodies (1 μg) were added into the reaction mixture was studied by immunoprecipitation and immunoblotting technique. Cells were preincubated with sodium butyrate for 2 days and then stimulated with different concentrations of IFN-α for 30 min. STAT1 protein was immunoprecipitated by a specific monoclonal antibody, resolved by SDS-PAGE and transferred to nitrocellulose membranes. The blots were then probed with an anti-phosphotyrosine antibody to investigate the phosphorylation status of STAT1. As demonstrated in Figure 2, phosphorylation of STAT1 was slightly induced by IFN-α in PLC/PRF/5 cells. After sodium butyrate pretreatment, IFN-α-induced phosphorylation of STAT1 was significantly enhanced, but sodium butyrate alone did not affect the phosphorylation status of STAT1. Because STAT1 is activated by phosphorylation, we next examined whether the DNA binding activity of STAT1 was also augmented by sodium butyrate in PLC/PRF/5 cells. It is well-known that IFN-α can regulate various gene expressions via two promoter motifs called the IFN-stimulated response element (ISRE) and the IFN-γ activation site (GAS), which bind the primary factor IFN-stimulated gene factor-3 (ISGF-3) and IFN-γ activation factor (GAF) respectively. The ISGF-3 complex contains phosphorylated STAT1, STAT2 and a p48 protein, and the GAF complex mainly contains phosphorylated STAT1:STAT1 homodimer or STAT1:STAT3 heterodimer. Because the foregoing experiments indicated that the expression of STAT2 was very low in PLC/PRF/5 cells and the expression of STAT1 was specifically activated by sodium butyrate, we therefore used a GAS oligonucleotide probe to evaluate the DNA binding activity of STAT1. Figure 3 shows that IFN-α activated GAF binding to GAS in PLC/PRF/5 cells in a dose-dependent manner. Interestingly, two gel shift complexes (GAF-1 and GAF-2) with similar characteristics were observed in our EMSA autoradiographs. Similar results have also been reported in HL60 and WISH cells after IFN-α stimulation, and these complexes have been shown to be STAT1:STAT3 (GAF-1) heterodimer and STAT1:STAT1 (GAF-2) homodimer respectively (Pelicano et al, 1997). The identity of these gel shift complexes as GAFs containing STAT1 was established by specific anti-STAT1 antibodies that neutralized the formation of GAF complexes but not by unrelated anti-c-Jun antibodies. Furthermore, unlabelled GAS oligonucleotides competed efficiently for GAF binding. Taken together, these data indicate that IFN-α-induced phosphorylation and activation of STAT1 is enhanced by sodium butyrate in PLC/PRF/5 cells.

### Sodium butyrate and IFN-α synergistically induce G1 growth arrest and apoptosis in PLC/PRF/5 cells

Because sodium butyrate-preincubated PLC/PRF/5 cells showed an increase in phosphorylation and activation of STAT1 after a short-term stimulation of IFN-α, it is of interest to determine if sodium butyrate can modulate the biological responses to IFN-α in these cells after a long-term culture. Thus, cells were treated with different combinations of drugs and cell cycle distribution was examined by flow cytometry. As shown in Table 2, the percentage of cells accumulated in G1 phase after treatment with 1 mM sodium butyrate or 2000 IU ml$^{-1}$ IFN-α for 48 h was 54% and 61%, respectively, while the percentage of G1 cells was 43% in untreated cells. However, 84% of cells were in G1 phase after incubation of PLC/PRF/5 cells with 2000 IU ml$^{-1}$ IFN-α and sodium butyrate simultaneously for 48 h. These results indicate that cell cycle progression was arrested in G1 phase. We further investigated the effect of these two drugs on the expression of cell cycle regulatory proteins in PLC/PRF/5 cells. As demonstrated in Figure 4, sodium butyrate (1 mM) or IFN-α (2000 IU ml$^{-1}$) alone slightly decrease the expression of cyclin D1, but simultaneous incubation of these two drugs completely inhibits the expression of cyclin D1. Furthermore, these two drugs synergistically activated the expression of p21$^{WAF-1}$, a specific cyclin-dependent kinase (CDK) inhibitor. Up-regulation of p21$^{WAF-1}$ and down-regulation of cyclin D1 leads to accumulation of hypophosphorylated form of retinoblastoma (Rb) proteins in these cells (Figure 4). Additionally, we found that cell viability was dramatically decreased after co-treatment of these two drugs for 3 days. After drug treatment for 48 or 72 h, cells were fixed and stained with the fluorochrome Hoechst 33258, and apoptotic cells with condensed chromatin fragments were observed under a fluorescence microscope and scored. The percentage of apoptoic nuclei in cells co-treated with sodium butyrate and IFN-α for 72 h is about 50%, but incubation with either drug alone did not significantly affect cell viability under the same experimental conditions (Table 3). Additionally, a characteristic DNA ladder pattern of DNA fragmentation was induced by co-treatment of these two drugs in PLC/PRF/5 cells (Figure 5). Conversely, incubation of sodium butyrate or IFN-α alone for 3 days did not induce DNA fragmentation in these cells. Thus, it is suggested that long-term treatment of sodium butyrate in combination with IFN-α may trigger apoptosis in PLC/PRF/5 hepatoma cells.

### DISCUSSION

The molecular mechanism for the deficiency of effects of IFNs in the inhibition of proliferation in hepatoma cells is not well defined, it is suggested that the insufficiency of expressions of important

 *© Cancer Research Campaign 1999*

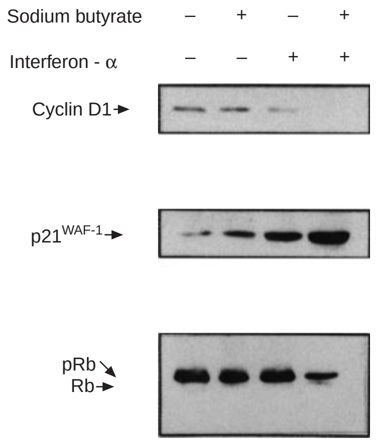

**Figure 4** Growth inhibition of PLC/PRF/5 hepatoma cells by sodium butyrate and IFN-α is associated with modulation of cyclin D1 and p21$^{WAF-1}$ expression and RB phosphorylation. Cells were treated with sodium butyrate (1 mM) or IFN-α (2000 IU $ml^{-1}$) or a combination of these two drugs for 2 days. Cells were harvested in a lysis buffer and analysis of expression of cell cycle regulatory proteins was performed by immunoblotting as described in Figure 1

**Table 3** Cotreatment of sodium butyrate and IFN-α induces apoptosis in PLC/PRF/5 cells

| | Apoptotic cells (% of total cell number) | |
|---|---|---|
| **Treatment** | **48 h** | **72 h** |
| Control (no addition) | 3 ± 2 | 8 ± 2 |
| Sodium butyrate (1 mM) | 4 ± 1 | 10 ± 3 |
| IFN-α (2000 IU $ml^{-1}$) | 8 ± 3 | 14 ± 2 |
| Sodium butyrate + IFN-α | 11 ± 5 | 48 ± 8 |

Cells were cultured in different combinations of drugs for 3 days. After that, cells were harvested, stained with Hoechst 33258 dye and counted under a fluorescence microscope. The values represent mean ± s.e.m. of three independent experiments.

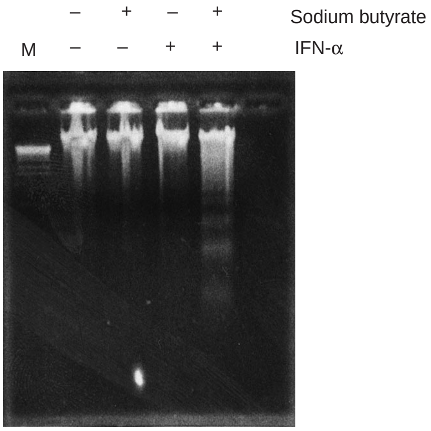

**Figure 5** Induction of a DNA ladder pattern of DNA fragmentation by sodium butyrate/IFN-α combination in PLC/PRF/5 cells. Cells were cultured in sodium butyrate (1 mM) or IFN-α or a combination of these two drugs for 3 days. Cells were collected in a lysis buffer and genomic DNA was extracted. DNA samples were subjected to agarose gel electrophoresis, stained with ethidium bromide and visualized under UV light signal transducers in the IFN-mediated pathway in human hepatoma cells may be responsive partially for this deficiency. Therefore, we tested whether enhancement of the expressions of these signal transduction molecules in human hepatoma cells may restore the response of these cells to IFNs. We found that the expression of STAT1, a transcription factor involved in the IFN-mediated signalling system, is specifically activated by sodium butyrate, and phosphorylation and activation of STAT1 induced by IFN-α is also significally enhanced by this drug. In agreement with our expectation, sodium butyrate augments the anti-proliferative effect of IFN-α. Pretreatment of IFN-unresponsive tumour cell lines with retinoic acid, another potent differentiation-inducing agent, also permitted growth inhibition by IFNs (Pelicano et al, 1997; Weihua et al, 1997). These studies indicated that augmentation of the expression of STAT1 by retinoic acid is an early step in the cooperative anti-tumour effects of retinoic acid and IFNs. In addition to in vitro study, clinical studies also demonstrated that IFN-α and 13-*cis*-retinoic acid produced substantial therapeutic benefits in patients with advanced squamous cell carcinomas of the skin and cervix (Lippman et al, 1992*a*, 1992*b*). Therefore, it is suggested that a rational combination of IFNs and differentiation-inducing agents may help to develop a new strategy for the treatment of human cancer. Our results support this idea and define a molecular basis for the synergy between IFNs and sodium butyrate in hepatoma growth inhibition. Furthermore, a recent study has reported that hepatitis B viral terminal protein shuts off IFN-induced gene expression by interfering with the formation of ISGF-3 and inhibited cellular responses to IFN-α and IFN-γ (Foster et al, 1991). The authors suggested that, in patients with chronic hepatitis B virus infection, an imbalance in HBV RNA and polymerase production may generate sufficient free polymerases or terminal proteins to give rise to IFN-resistant cells. Clinically, this may be one of the mechanisms by which patients develop resistance to IFN therapy during chronic viral infection. Considered together, our results are of clinical importance in the treatment of viral hepatitis and liver cancer.

Another important observation in our study is the synergy between IFN-α and sodium butyrate in the induction of p21$^{WAF-1}$ expression. This protein was originally identified as a CDK2-interactive protein by using the yeast two-hybrid technique and was found to be a CDK inhibitor (Harper et al, 1993). Subsequently, accumulating data suggested that p21$^{WAF-1}$ is a critical mediator of p53-mediated G1 phase arrest (El-Deiry et al, 1993, 1994). Many human cancer cells (including hepatoma cells) harbour mutant p53 proteins, these cells do not arrest in the G1 phase and do not accumulate p21$^{WAF-1}$ after DNA-damaging agent treatment, thus resulting in de-regulated cell growth control. It has been demonstrated that p21$^{WAF-1}$ expression can be induced via p53-independent pathways and enforced expression of this protein in cancer cells harbouring mutant p53 proteins may induce G1 phase arrest and inhibit tumour growth (Xiong et al, 1993; Harper et al, 1995). Recent study showed that IFNs might trigger expression of p21$^{WAF-1}$ via STAT1 sites (Chin et al, 1996). Thus, sodium butyrate may act via up-regulation of STAT1 expression to enhance IFN-α-induced p21$^{WAF-1}$ expression in PLC/PRF/5 cells. However, it is also possible that sodium butyrate may act via Sp1 sites independently to activate p21$^{WAF-1}$ expression as reported recently (Nakano et al, 1997). Study of the collaboration between different consensus sites in p21$^{WAF-1}$ promoter will be helpful for the understanding of regulation of p21$^{WAF-1}$ expression in cells receiving different stimuli or insults.

*© Cancer Research Campaign 1999*

We also found that apoptosis of PLC/PRF/5 cells could be induced by a combination of sodium butyrate and IFNs. Although the mechanism by which these two drugs trigger apoptotic cell death is unclear, recent works demonstrated that activation of the STAT signalling pathway may cause expression of caspase-1 and induction of apoptosis (Chin et al, 1997). Caspase-1, originally known as interleukin-1β-converting enzyme (ICE), is a member of the mammalian ICE protease family which plays an important role in the executing phase of apoptosis and is homologous to the *C. elegans* protein ced-3 (Kumar, 1995). These proteases are thought to induce cell death by cleaving vital cellular proteins (such as nuclear lamins, poly(ADP)-ribose polymerase, U1-associated 70 kDa protein etc.). It has also been identified that several caspases may be activated simultaneously or sequentially by different apoptotic signals. Whether ICE/ICE-like proteases are involved in the apoptotic signalling pathway induced by sodium butyrate and IFN-α needs further investigation. In summary, our results demonstrate that the combination of sodium butyrate and IFN-α is highly effective in the inhibition of hepatoma cell proliferation in vitro and may lead to additional therapeutical approaches against both liver cancer and viral infectious diseases. Furthermore, due to its low cytotoxicity, sodium butyrate is considered to be a better therapeutical adjuvant than other differentiation-inducing agents (such as retinoic acid or dimethyl sulphoxide).

### ACKNOWLEDGEMENTS

The authors thank the Hoffmann-La Roche Inc. for providing recombinant interferon-α used in this study.

### REFERENCES

Alexander GJ, Brahm J, Fagan EA, Smith HM, Daniels HM, Eddleston AL and Williams R (1987) Loss of HBsAg with interferon therapy in chronic hepatitis virus infection. *Lancet* **2**: 66–69

Boue F, Pastran Z, Spielmann M, Chevalier TL, Subirana R, Sevin D, Paoletti C, Brandely M, Avril MF, Sancho-Garnier H and Tursz T (1990) A phase I trial with recombinant interferon γ (Rousell UCLAF) in advanced cancer patients. *Cancer Immunol Immunother* **32**: 67–70

Chin YE, Kitagawa M, Su WCS, You ZH, Iwamoto Y and Fu XY (1996) Cell growth arrest and induction of cyclin-dependent kinase inhibitor p21$^{WAF-1/CIP1}$ mediated by STAT1. *Science* **272**: 719–722

Chin YE, Kitagawa M, Kuida K, Flavell RA and Fu XY (1997) Activation of the STAT signaling pathway can cause expression of caspase 1 and apoptosis. *Mol Cell Biol* **17**: 5328–5337

Darnell JE Jr (1997) STATs and gene regulation. *Science* **277**: 1630–1635

Decker T, Lew DJ and Darnell JE Jr (1991) Two distinct alpha-interferon dependent signal transduction pathways may contribute to activation of transcription to the guanylate-binding protein gene. *Mol Cell Biol* **13**: 5147–5153

El-Deiry W, Tokino T, Velculescu VE, Levy DB, Parsons R, Trent JM, Lin D, Mercer WE, Kinzler KW and Vogelstein B (1993) WAF-1, a potential mediator of p53 tumor suppression. *Cell* **75**: 817–825

El-Deiry W, Harper JW, O'Connor PM, Velculescu VE, Canman CE, Jackman J, Pietenpol JA, Burrell M, Hill DE, Wang Y, Wiman KG, Mercer WE, Kastan MB, Kohn KW, Elledge SJ, Kinzler KW and Vogelstein B (1994) WAF1/CIP1 is induced in p53-mediated G1 arrest and apoptosis. *Cancer Res* **54**: 1169–1174

Foster GR, Ackrill AM, Goldin RD, Kerr IM, Thomas HC and Stark GR (1991) Expression of the terminal protein region of hepatitis B virus inhibits cellular responses to interferon α and γ double-stranded RNA. *Proc Natl Acad Sci USA* **88**: 2888–2892

Goto I, Yamamoto-Yamagichi Y and Honma Y (1996) Enhancement of sensitivity of human lung adenocarcinoma cells to growth-inhibitory activity of interferon α by differentiation-inducing agents. *Br J Cancer* **74**: 546–554

Gutterman JU (1994) Cytokine therapeutics: lessons from interferon α. *Proc Natl Acad Sci USA* **91**: 1198–1205

Harper JW, Adami GR, Wei N, Keyomarsi K and Elledge SJ (1993) The p21 Cdk-interacting protein Cip1 is a potent inhibitor of G1 cyclin-dependent kinases. *Cell* **75**: 805–816

Harper JW, Elledge SJ, Keyomarsi K, Dynlacht B, Tsai LH, Zhang P, Dobrowolski S, Bai C, Connell-Crowley L, Swindell E, Fox MP and Wei N (1995) Inhibition of cyclin-dependent kinases by p21. *Mol Biol Cell* **6**: 387–400

Hung WC and Chuang LY (1996) Induction of apoptosis by sphingosine-1-phosphate in human hepatoma cells is associated with enhanced expression of bax gene product. *Biochem Biophys Res Commun* **229**: 11–15

Hung WC, Yang ML, Chang CC, Tsai JH and Chuang LY (1995) Differential regulation of EGF production, EGF receptor binding, and cellular growth by sodium butyrate in Hep3B and PLC/PRF/5 human hepatoma cells. *Inter J Oncol* **7**: 1089–1093

Kolla V, Lindner DJ, Weihua X, Borden EC and Kalvakolanu DV (1996) Modulation of interferon-inducible gene expression by retinoic acid: up-regulation of STAT1 protein in interferon-unresponsive cells. *J Biol Chem* **271**: 10508–10514

Korenman J, Baker B, Waggoner J, Everhart JE, Di-Bisceglie AM and Hoofnagle JH (1991) Long-term remission of chronic hepatitis B after alpha-interferon therapy. *Ann Intern Med* **114**: 629–634

Kumar S (1995) ICE-like proteases in apoptosis. *Trends Biochem Sci* **20**: 198–202

Lai CL, Wu PC, Lok ASF, Lin HJ, Ngan H, Lau JYN, Chung HT, Ng MMT, Yeoh EK and Arnold M (1989) Recombinant interferon α2 is superior to doxorubin for inoperable hepatocellular carcinoma: a prospective randomised trial. *Br J Cancer* **60**: 928–933

Lee CK, Bluyssen HAR and Levy DE (1997) Regulation of interferon-α responsiveness by the duration of janus kinase activity. *J Biol Chem* **272**: 21872–21877

Lippman SM, Parkinson DR, Itri LM, Weber RS, Schantz SP, Ota DM, Schusterman MA, Krakoff IH, Gutterman JU and Hong WK (1992*a*) 13-*cis*-retinoic acid and interferon α-2a: effective combination therapy for advanced squamous cell carcinoma of the skin. *J Natl Cancer Inst* **84**: 235–241

Lippman SM, Kavanagh JJ, Pareds-Espinoza M, Delgadillo-Madrueno P, Paredes-Casillas P, Hong WK, Holdener E and Krakoff IH (1992*b*) 13-*cis*-retinoic acid plus interferon α-2a: highly active systemic therapy for squamous cell carcinoma of the cervix. *J Natl Cancer Inst* **84**: 241–245

Muller M, Laxton C, Briscoe J, Schindler C, Improta T, Darnell JE Jr, Stark GR and Kerr IM (1993) Complementation of a mutant cell line: central role of the 91 kDa polypeptide of ISGF3 in the interferon-alpha and -gamma signal transduction pathways. *EMBO J* **12**: 4221–4228

Nair PV, Tong MJ, Kempf R, Co R, Lee SD and Venturi CL (1985) Clinical serologic and immunological effects of human leukocyte interferon in HBsAg-positive primary hepatocellular carcinoma. *Cancer* **56**: 1018–1023

Nakano K, Mizuno T, Sowa Y, Orita T, Yoshino T, Okuyam Y, Fujita T, Ohtani-Fujita N, Matsukawa Y, Tokino T, Yamagishi H, Oka T, Nomura H and Sakai T (1997) Butyrate activates the WAF1/CIP1 gene promoter through Sp1 sites in a p53-negative human colon cancer cell line. *J Biol Chem* **272**: 22199–22206

Pelicano L, Li F, Schindler C and Chelbi-Alix MK (1997) Retinoic acid enhances the expression of interferon-induced proteins: evidence for multiple mechanisms of action. *Oncogene* **15**: 2349–2359

Pellegrini S and Dusanter-Fourt I (1997) The structure, regulation and function of the Janus kinases (JAKs) and the signal transducers and activators of transcription (STATs). *Eur J Biochem* **248**: 615–633

Sachs E, Di-Biscegle AM, Dusheiko GM, Song E, Lyons SF, Schoub BD and Kew MC (1985) Treatment of hepatocellular carcinoma with recombinant leucocyte interferon: a pilot study. *Br J Cancer* **52**: 105–109

Tur-Kaspa R, Teicher L, Laub O, Itin A, Dagan D Bloom BR and Shafritz DA (1990) Alpha interferon suppresses hepatitis B virus enhancer activity and reduces viral gene transcription. *Virol* **64**: 1821–1824

Weihua X, Kolla V and Kalvakolanu DV (1997) Modulation of interferon action by retinoids: induction of murine STAT1 gene expression by retinoic acid. *J Biol Chem* **272**: 9742–9748

Xiong Y, Hannon GJ, Zhang H, Casso D, Kobayashi R and Beach D (1993) p21 is a universal inhibitor of cyclin kinases. *Nature* **366**: 701–704

Xu B, Grander D, Sangfelt O and Einhorn S (1994) Primary leukemia cells resistant to α-interferon in vitro are defective in the activation of the DNA-binding factor interferon-stimulated gene factor 3. *Blood* **84**: 1942–1949

Yamamoto K, Quelle FW, Thierfelder WE, Kreider BL, Gilbert DJ, Jenkins NA, Copeland NG, Silvennoinen O and Ihle JN (1994) Stat4, a novel gamma interferon activation site-binding protein expressed in early myeloid differentiation. *Mol Cell Biol* **14**: 4342–4349

Zhang P and McLachlan A (1994) Differentiation-specific transcriptional regulation of the hepatitis B virus nucleocapsid gene in human hepatoma cell lines. *Virology* **202**: 430–440

© Cancer Research Campaign 1999

## References

[PDF_00533] Alexander G. J., Brahm J., Fagan E. A., Smith H. M., Daniels H. M., Eddleston A. L., Williams R. (1987). Loss of HBsAg with interferon therapy in chronic hepatitis B virus infection.. Lancet.

[PDF_00536] Boue F., Pastran Z., Spielmann M., Le Chevalier T., Subirana R., Sevin D., Paoletti C., Brandely M., Avril M. F., Sancho-Garnier H. (1990). A phase I trial with recombinant interferon gamma (Roussel UCLAF) in advanced cancer patients.. Cancer Immunol Immunother.

[PDF_00543] Chin Y. E., Kitagawa M., Kuida K., Flavell R. A., Fu X. Y. (1997). Activation of the STAT signaling pathway can cause expression of caspase 1 and apoptosis.. Mol Cell Biol.

[PDF_00540] Chin Y. E., Kitagawa M., Su W. C., You Z. H., Iwamoto Y., Fu X. Y. (1996). Cell growth arrest and induction of cyclin-dependent kinase inhibitor p21 WAF1/CIP1 mediated by STAT1.. Science.

[PDF_00546] Darnell J. E. (1997). STATs and gene regulation.. Science.

[PDF_00547] Decker T., Lew D. J., Darnell J. E. (1991). Two distinct alpha-interferon-dependent signal transduction pathways may contribute to activation of transcription of the guanylate-binding protein gene.. Mol Cell Biol.

[PDF_00558] Foster G. R., Ackrill A. M., Goldin R. D., Kerr I. M., Thomas H. C., Stark G. R. (1991). Expression of the terminal protein region of hepatitis B virus inhibits cellular responses to interferons alpha and gamma and double-stranded RNA.. Proc Natl Acad Sci U S A.

[PDF_00562] Goto I., Yamamoto-Yamaguchi Y., Honma Y. (1996). Enhancement of sensitivity of human lung adenocarcinoma cells to growth-inhibitory activity of interferon alpha by differentiation-inducing agents.. Br J Cancer.

[PDF_00565] Gutterman J. U. (1994). Cytokine therapeutics: lessons from interferon alpha.. Proc Natl Acad Sci U S A.

[PDF_00567] Harper J. W., Adami G. R., Wei N., Keyomarsi K., Elledge S. J. (1993). The p21 Cdk-interacting protein Cip1 is a potent inhibitor of G1 cyclin-dependent kinases.. Cell.

[PDF_00570] Harper J. W., Elledge S. J., Keyomarsi K., Dynlacht B., Tsai L. H., Zhang P., Dobrowolski S., Bai C., Connell-Crowley L., Swindell E. (1995). Inhibition of cyclin-dependent kinases by p21.. Mol Biol Cell.

[PDF_00573] Hung W. C., Chuang L. Y. (1996). Induction of apoptosis by sphingosine-1-phosphate in human hepatoma cells is associated with enhanced expression of bax gene product.. Biochem Biophys Res Commun.

[PDF_00580] Kolla V., Lindner D. J., Xiao W., Borden E. C., Kalvakolanu D. V. (1996). Modulation of interferon (IFN)-inducible gene expression by retinoic acid. Up-regulation of STAT1 protein in IFN-unresponsive cells.. J Biol Chem.

[PDF_00626] Kolla V., Weihua X., Kalvakolanu D. V. (1997). Modulation of interferon action by retinoids. Induction of murine STAT1 gene expression by retinoic acid.. J Biol Chem.

[PDF_00584] Korenman J., Baker B., Waggoner J., Everhart J. E., Di Bisceglie A. M., Hoofnagle J. H. (1991). Long-term remission of chronic hepatitis B after alpha-interferon therapy.. Ann Intern Med.

[PDF_00587] Kumar S. (1995). ICE-like proteases in apoptosis.. Trends Biochem Sci.

[PDF_00588] Lai C. L., Wu P. C., Lok A. S., Lin H. J., Ngan H., Lau J. Y., Chung H. T., Ng M. M., Yeoh E. K., Arnold M. (1989). Recombinant alpha 2 interferon is superior to doxorubicin for inoperable hepatocellular carcinoma: a prospective randomised trial.. Br J Cancer.

[PDF_00592] Lee C. K., Bluyssen H. A., Levy D. E. (1997). Regulation of interferon-alpha responsiveness by the duration of Janus kinase activity.. J Biol Chem.

[PDF_00600] Lippman S. M., Kavanagh J. J., Paredes-Espinoza M., Delgadillo-Madrueño F., Paredes-Casillas P., Hong W. K., Holdener E., Krakoff I. H. (1992). 13-cis-retinoic acid plus interferon alpha-2a: highly active systemic therapy for squamous cell carcinoma of the cervix.. J Natl Cancer Inst.

[PDF_00595] Lippman S. M., Parkinson D. R., Itri L. M., Weber R. S., Schantz S. P., Ota D. M., Schusterman M. A., Krakoff I. H., Gutterman J. U., Hong W. K. (1992). 13-cis-retinoic acid and interferon alpha-2a: effective combination therapy for advanced squamous cell carcinoma of the skin.. J Natl Cancer Inst.

[PDF_00603] Müller M., Laxton C., Briscoe J., Schindler C., Improta T., Darnell J. E., Stark G. R., Kerr I. M. (1993). Complementation of a mutant cell line: central role of the 91 kDa polypeptide of ISGF3 in the interferon-alpha and -gamma signal transduction pathways.. EMBO J.

[PDF_00607] Nair P. V., Tong M. J., Kempf R., Co R., Lee S. D., Venturi C. L. (1985). Clinical, serologic, and immunologic effects of human leukocyte interferon in HBsAg-positive primary hepatocellular carcinoma.. Cancer.

[PDF_00611] Nakano K., Mizuno T., Sowa Y., Orita T., Yoshino T., Okuyama Y., Fujita T., Ohtani-Fujita N., Matsukawa Y., Tokino T. (1997). Butyrate activates the WAF1/Cip1 gene promoter through Sp1 sites in a p53-negative human colon cancer cell line.. J Biol Chem.

[PDF_00614] Pelicano L., Li F., Schindler C., Chelbi-Alix M. K. (1997). Retinoic acid enhances the expression of interferon-induced proteins: evidence for multiple mechanisms of action.. Oncogene.

[PDF_00617] Pellegrini S., Dusanter-Fourt I. (1997). The structure, regulation and function of the Janus kinases (JAKs) and the signal transducers and activators of transcription (STATs).. Eur J Biochem.

[PDF_00620] Sachs E., Di Bisceglie A. M., Dusheiko G. M., Song E., Lyons S. F., Schoub B. D., Kew M. C. (1985). Treatment of hepatocellular carcinoma with recombinant leucocyte interferon: a pilot study.. Br J Cancer.

[PDF_00623] Tur-Kaspa R., Teicher L., Laub O., Itin A., Dagan D., Bloom B. R., Shafritz D. A. (1990). Alpha interferon suppresses hepatitis B virus enhancer activity and reduces viral gene transcription.. J Virol.

[PDF_00629] Xiong Y., Hannon G. J., Zhang H., Casso D., Kobayashi R., Beach D. (1993). p21 is a universal inhibitor of cyclin kinases.. Nature.

[PDF_00631] Xu B., Grandér D., Sangfelt O., Einhorn S. (1994). Primary leukemia cells resistant to alpha-interferon in vitro are defective in the activation of the DNA-binding factor interferon-stimulated gene factor 3.. Blood.

[PDF_00634] Yamamoto K., Quelle F. W., Thierfelder W. E., Kreider B. L., Gilbert D. J., Jenkins N. A., Copeland N. G., Silvennoinen O., Ihle J. N. (1994). Stat4, a novel gamma interferon activation site-binding protein expressed in early myeloid differentiation.. Mol Cell Biol.

[PDF_00638] Zhang P., McLachlan A. (1994). Differentiation-specific transcriptional regulation of the hepatitis B virus nucleocapsid gene in human hepatoma cell lines.. Virology.

[PDF_00553] el-Deiry W. S., Harper J. W., O'Connor P. M., Velculescu V. E., Canman C. E., Jackman J., Pietenpol J. A., Burrell M., Hill D. E., Wang Y. (1994). WAF1/CIP1 is induced in p53-mediated G1 arrest and apoptosis.. Cancer Res.

[PDF_00550] el-Deiry W. S., Tokino T., Velculescu V. E., Levy D. B., Parsons R., Trent J. M., Lin D., Mercer W. E., Kinzler K. W., Vogelstein B. (1993). WAF1, a potential mediator of p53 tumor suppression.. Cell.

